# Can pelvic diameter measurement have an effect on surgical outcomes in radical cystectomy?

**DOI:** 10.1186/s12894-023-01277-8

**Published:** 2023-06-07

**Authors:** Anil Erdik, Haci Ibrahim Cimen, Osman Kose, Omer Faruk Ates, Onur Taydas, Deniz Gul, Hasan Salih Saglam

**Affiliations:** 1 Department of Urology, Sadıka Sabancı Hospital, 54050 Sakarya, Turkey; 2 Department of Urology, Sakarya University, School of Medicine, 54100 Sakarya, Turkey; 3 Department of Radiology, Sakarya University, School of Medicine, 54100 Sakarya, Turkey

**Keywords:** Charlson comorbidity score, Pelvis, Postoperative complication, Radical cystectomy, Surgical margin

## Abstract

**Objective:**

To determine the effectiveness of pelvis diameters in determining postoperative outcomes in men who underwent open radical cystectomy + urinary diversion, it is aimed to predict the factors that may affect the operative difficulty and possible surgical outcomes before the operation.

**Methods:**

A total of 79 radical cystectomy patients operated in our institution with preoperative computed tomography (CT) were included the study. Pelvic dimensions; symphysis angle (SA), upper conjugate, lower conjugate, pelvic depth, apical depth (AD), interspinous distance (ISD), bone femoral width and soft tissue width were measured by preoperative CT. ISD index were defined as ISD/AD. Postoperative outcomes and indicators of operative difficulty were recorded. Regression analyses were used to predict perioperative and postoperative outcomes.

**Results:**

Total of 96 complications were observed in 52 of the 79 patients in ninety days (65,8%) with a mean age of 68.25 years. There were significant correlations between SA and body mass index (BMI) with operative time (*p* = 0.006, *p* < 0.001; respectively). For estimated blood loss, there were significant correlations between preoperative hematocrit (*p* = 0,031). Analysis of multivariate logistic regression revealed that higher Charlson comorbidity index (CCI) and BMI were found to be significant predictors for major complications while CCI, pathological T stage and ISD index are prominent predictors for surgical margin positivity.

**Conclusions:**

Pelvic dimensions are not significant with minor or major complications. However, operative time may be associated with SA. Also, narrow and deep pelvis may increase the risk of positive surgical margins.

**Supplementary Information:**

The online version contains supplementary material available at 10.1186/s12894-023-01277-8.

## Introduction

While 70% of patients with newly diagnosed bladder cancer (BC) present with the disease confined to the mucosa (stage Ta, carcinoma in situ) or submucosa (T1), the remaining cases often include BC has invaded the muscle [1]. Today, as in the past, bilateral extended pelvic lymph node dissection and urinary diversion surgery in tandem with radical cystectomy (RC) is the accepted gold standard treatment for muscle-invasive BC [2]. However, perioperative morbidity and mortality increase and continence rates deteriorate after orthotopic urinary diversion in patients who have undergone RC, especially among the elderly [3].

Complications after BC surgery can be classified as early or late and major or minor. A prior study revealed that the most common complications of BC surgery regardless of the patient's age, are ileus and infection-related complications (e.g. fever and pyelonephritis) [4]. Complications can be listed as non-specific (e.g. atelectasis or myocardial infarction), diversion related (e.g. urinary fistula, stoma-related stricture and ureteral stricture), or neurological (e.g. delirium) [5]. Despite surgical and medical advances, RC and urinary diversion are associated with significant postoperative mortality ranging from 4 to 11% within 90 days after surgery [6].

Anatomical variations in pelvic dimensions may play an important role in likelihood of developing operative difficulties and complications during an RC procedure such as radical prostatectomy (RP). These variations may be the cause of a high percentage of negative postoperative outcomes (e.g. major complication or positive surgical margin etc.) and a technically challenging operation. A diagnostic model may be useful in predicting RC needed to predicting RC outcomes.

Thin-section computed tomography (CT) is used to determine a patient’s pelvic dimensions before a RC. The potential advantages of this method of imaging as opposed to others is increased speed, a reduction in distortions due to movement and the need for less contrast material, which reduces the risk of nephrotoxicity fast imaging, image distortions due to movement are reduced, and the amount of contrast material decreases significantly thanks to the speed; reduce the risk of nephrotoxicity.

A number of studies have on the relationship between anatomical pelvic variations and perioperative outcomes and postoperative complications of RP [7–11]. However, little published information exists on the effects of pelvic dimensions on RC outcomes [12]. To date, no research has shown a link between pelvic dimensions and RC postoperative outcomes. Because the impact of pelvic dimensions on RC postoperative outcomes is not clear, this diagnostic study examined the relationship between pelvic dimensions and the development of operative difficulties and negative postoperative outcomes. This was done through a retrospective examination of the postoperative complications of patients who underwent RC and urinary diversion operations due to high-risk and muscle-invasive BC.

## Materials and methods

### Data collection, patient characteristics and clinical variables

This single centered, retrospective study was approved by the Institutional Review Board of the Ethics Committee of Sakarya University College of Medicine (Approval Number through 00384, 4 June 2020). The study followed the ethical principles of the Declaration of Helsinki. Informed consent was obtained from all subjects when they were admitted to the hospital.

Inclusion criteria for the study participants included having undergone RC after a diagnosis of high risk or invasive BC in our tertiary referral hospital clinic from September 2014 to March 2020. The first step in the study was to collect data on the paticipants retrospectively, which resulted in the collection of data on 129. Of these, 22 patients lacked computed tomography images taken before the operation, so the data on these patients were excluded from the study. Apical depth (AD) could not be evaluated in 28 female patients, resulting in their exclusion. Ultimately, the study included data from 79 male patients.

The following clinicopathological variables were recorded: complications observed intraoperatively, operative time (OT), estimated blood loss (EBL), transfusion rate and type of diversion. The following postoperative variables were recorded: pathological T stage identified in the pathology report, positive surgical margin (PSM), lymph node involvement, complication rates according to Clavien-Dindo (CD) classification, length of hospital stay (HS), readmission to the hospital within 30 days and mortality within 90 days.

### Surgical techniques and follow-up

Each patient had undergone an open radical cystectomy, an extended pelvic lymphadenectomy and an ileal conduit following the standard procedures. Postoperative routine, daily examinations and laboratory and imaging follow-ups had been conducted as standard. The patients had been seen for follow-up care at one and three months after the RC. Readmission status up to 30 days and mortality and complications observed up to the 90th postoperative day were recorded according to the CD scoring system, in which, major complications are defined as grades 3–5 and minor or no complications are defined as grades 0–2.

### Computed tomography imaging and pelvic dimensions

Pelvic dimensions were measured by transferring preoperative thin-section CT images of the patients’ pelvises to the KarPACS viewer 3.1.9.314 version programme (Mersin, Turkey, 2020). The images were reviewed by two radiologists blinded to the patients’ characteristics. In cases of disagreement the radiologists sought to reach, a consensus.

**Fig. 1:**
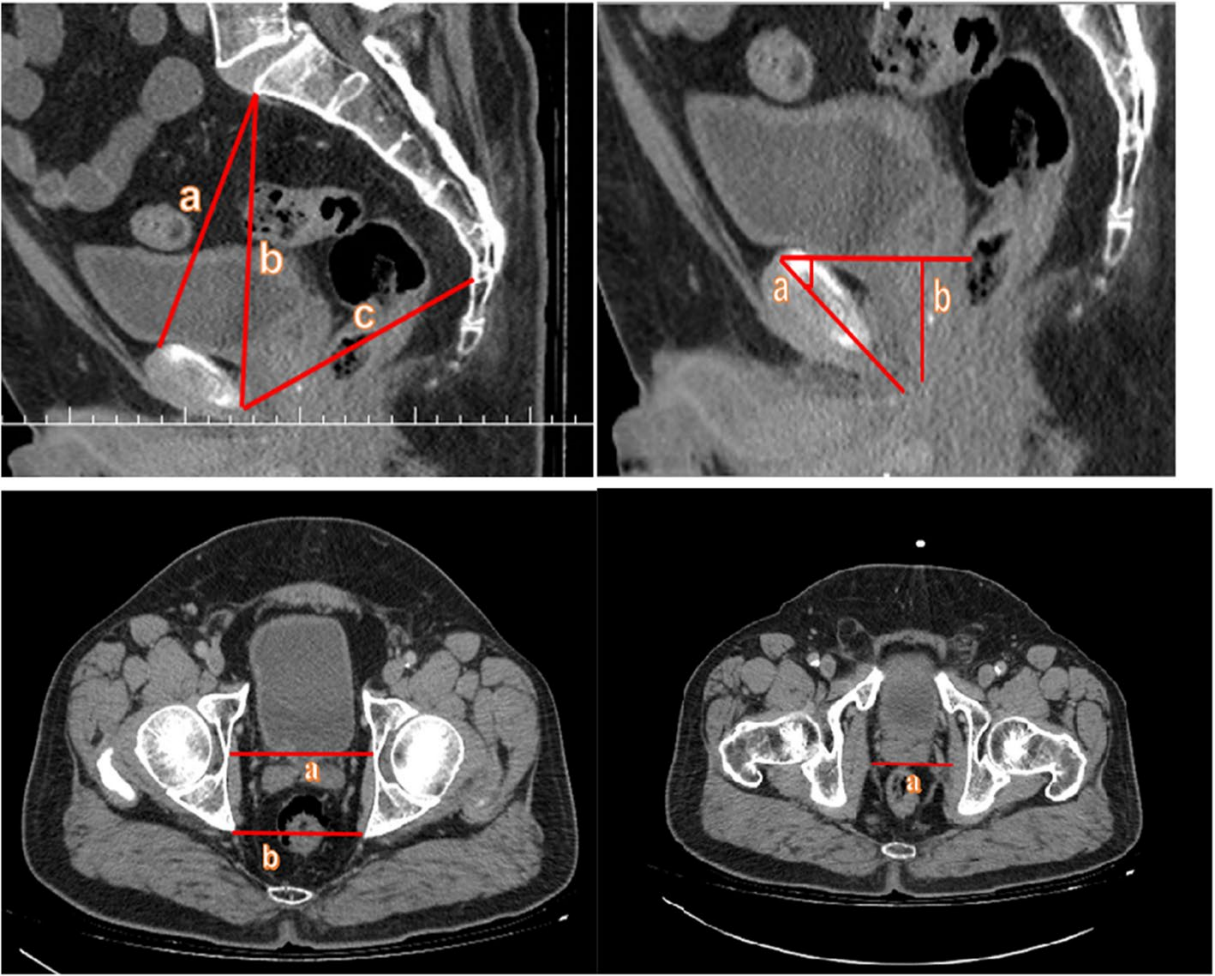
Upper left A) the upper conjugate (UC) was defined as the distance from the innermost aspect of the top of the symphysis pubis to the sacral promontory on the mid-sagittal plane; B) the pelvic depth (PD) was described as the distance between the promontorium and the lower symphysis pubis; C) The lower conjugate (LC) was defined as the distance from the lower symphysis pubis to the sacrococcygeal junction determined on the mid-sagittal plane. Upper right A) The symphysis angle (SA) was described as the angle between the long axis of the symphysis and the horizontal midsagittal plane. B) The apical depth (AD) was described as the distance between the highest point of the symphysis to the prostatic apex down left. Down left A) Bone femoral width (BFW) was mesasured as the bony width of the pelvis at the mid femoral head level. B) Interspinous distance (ISD) was measured on the axial plane between the tips of the ischial spines. Downright A) The soft tissue width (SW) was measured as the narrowest distance between the levator muscles on the axial CT images

The following pelvic parameters were used: the upper conjugate (UC) was defined as the distance from the innermost aspect of the top of the symphysis pubis to the sacral promontory on the midsagittal plane while distance from the lower symphysis pubis to the sacrococcygeal junction on the mid-sagittal plane was defined as the lower conjugate (LC) [12]. The distance between between the promontory and the lower symphysis pubis described the pelvic depth (PD) [12]. The symphysis angle (SA) was described as the angle between the long axis of the symphysis and the horizontal midsagittal plane while the distance between the highest point of the symphysis to the prostatic apex was described as the apical depth (AD) [12]. The interspinous distance (ISD) was measured on the axial plane between the tips of the ischial spines. Bony femoral width (BFW) was measured as of the pelvis at the mid-femoral head level on the axial plane while the narrowest distance between the levator muscles was measured as soft tissue width (SW) [12]. All pelvic dimensions in this study are shown in ‘Fig. 1’. BFW, ISD and SW indexes were described as BFW/AD, ISD/AD and SW/AD, respectively [12]. Following the measurement of the patients’ pelvic dimensions the effects of these measurements on surgical and postoperative outcomes were assessed.

### Statistical analysis

Age, BMI, prostate volume, Charlson comorbidity index (CCI) score, pathological stage, ASA score, preoperative albumin, creatinine and hematocrit (HCT) were analysed for correlation with operative difficulty, indicated by OT, EBL and length of HS. The association of perioperative outcomes (complications or major complications, operative difficulty, mortality and PSM) with age, BMI, prostate volume, CCI score groups (i.e. < 5 indicates low or moderate comorbidity and ≥ 5 indicates high comorbidity), ASA score, preoperative albumin, creatinine, HCT and extravesical disease were analysed with using Pearson’s chi-squared test, Fisher's exact test, independent samples t-test and Mann–Whitney U tests. For categorical variables, Pearson chi-squared or Fisher's exact test were used. For continuous variables, in cases where normality criteria existed, an independent samples t-test was performed; otherwise, the Mann–Whitney U test was used. Independent predictors of operative difficulty and postoperative outcomes were investigated using multivariate linear and logistic regression analyses. Age, BMI, ASA score, prostate volume, CCI score, pathological stage and pelvic dimensions were included in the regression analyses.

An intraclass correlation test was used to evaluate the concordance of multiple measurements obtained from the same patient, and good agreement was obtained (intraclass correlation coefficient > 0.750, *p* < 0.001). Statistical significance was assumed at *p* < 0.05. The standard effect size was set to 0.54 with an sample of 79 patients and a 5% standard error (a) margin. Based on these calculations, the study power was found to be 99% effect (1-b) to providing sufficient data. Statistical significance was considered at *p* < 0.05. Statistical analyses were performed using IBM SPSS Statistics for Windows (version 21.0, IBM Corp., Armonk, NY, USA) and G*Power for Windows (version 3.1.9.7, Heinrich-Heine-Universität, Düsseldorf, Germany).

## Results

### Patients characteristics and complication types

The patients’ mean age was 68.25 ± 9.71 years. Clinical features, preoperative and postoperative results, pelvic dimensions and pathological evaluations of the patients are shown in Table 1. While complications were observed in 8 patients perioperatively (e.g. rectum injury, etc.), a total of 96 complications were observed in 52 of the 79 patients (65.8%) within 90 days of RC. The most common complication category was complications of the gastrointestinal system, appearing in 35.4% (n:28) of the cases, followed by infectious complications with 27.8% (n:22) and wound/skin complications with 21.5% (n:17). Ileus was the most common complication in 21 patients (26.5%) (Supplementary Table 1).

**Table 1:** Clinical features, preoperative and postoperative findings, pelvic dimensions and pathological results

	**Mean ± SD**	**Median (Min–Max)**
Age ( year)	68.25 ± 9.71	69 (29–93)
BMI ( kg/m^2^)	25.96 ± 3.19	26.50 (19–35,1)
Prostate volume (cc)	47.94 ± 25.40	42 (20–168)
HGB (g/dL)	12.01 ± 1.58	12 (9–15.60)
HCT (%)	35.81 ± 4.60	35.60 (27–46.80)
Creatinine (mg/dL)	1.22 ± 0.53	1.10 (0.6–3.5)
Albumin (g/L)	3.52 ± 0.61	3.6 (2.3–4.5)
Operation time (min)	322 ± 76.96	320 (169–551)
Hospital stay (day)	11.93 ± 6.58	11 (4–49)
Estimated blood loss (mL)	1356.96 ± 514.88	1250 (500–3000)
**Pelvic Dimensions**		
UC	10.17 ± 0.79	10.14 (8.29–11.90)
LC	11.02 ± 0.89	11.20 (9.11–13.07)
PD	11.81 ± 0.85	11.80 (9.71–13.49)
AD	3.19 ± 0.40	3.12 (2.33–3.97)
SA	41.99 ± 5.36	41.09 (29.89–57.64)
ISD	8.93 ± 0.65	8.81 (7.33–10.60)
BFW	10.15 ± 0.53	10.09 (9.07–11.80)
SW	4.60 ± 0.36	4.63 (3.54–5.79)
ISD index	2.86 ± 0.41	2.85 (2.02–4.04)
BFW index	3.25 ± 0.44	3.21 (2.45–4.85)
SW index	1.47 ± 0.22	1.46 (1.03–2.33)
	**n**	**%**
**ASA Score**		
2	35	44.3
3	43	54.4
4	1	1.3
**Charlson score**		
< 5	54	68.4
≥ 5	25	31.6
**Pathological stage**		
pTa + PTcis	10	12.7
pT1	4	5.1
pT2	22	27.8
pT3	24	30.4
pT4	17	21.5
Excluding TCC	2	2.5
**Lymph node**		
N0	52	65.8
N1	17	21.5
N2	10	12.7
**Clavien-Dindo classification**		
Minor Complication (Grade 0–2)	30	38
Major Complication (Grade 3–5)	22	27.8
90-day mortality (Grade 5 complication)	11	13.9
Prior abdominal surgery	7	8.9
Perioperative complication	8	10.1
Postoperative complication	52	65.8
Perioperative transfusion	25	31.6
Surgical margin positivity	11	13.9

*SD* Standard deviation, *ASA* American Society of Anesthesiologists, *BMI* Body Mass Index, *CİS* Carsinoma in situ, *HGB* Hemoglobin, *HCT* Hematocrit, *UC* Upper conjugate, *LC* Lower conjugate, *PD* Pelvic depth, *AD* Apical depth, *SA* Symphysis angle, *ISD* Interspinous distance, *BFW* Bony femoral width, *SW* Soft tissue width, *TCC* Transitional cell carcinoma

Major complications were observed in 22 patients (27.8%), of which 7 (8.9%) were grade 3 and 4 (5.1%) were grade 4. Eleven patients (13.9%) died within 90 days after the surgery, and 11 died of cardiopulmonary events (myocardial infarction, pulmonary embolism or respiratory failure). Eleven patients (13.9%) had PSMs.

### Relationship between pelvic dimensions and perioperative outcomes

Among the operative difficulty parameters, OT was found to be correlated with BMI (*p*: < 0.001, r:0.620) and SA (p:0.011, r:-0.285). High SA values were related to short OT. EBL was found to be correlated with preoperative HCT (p:0.022, r:0.257) and LC (p:0.047, r:-0.225). Less EBL was related to higher LC values. No correlation was found between HS and other variables, including pelvic dimensions, BMI and CCI score. HS was not included in the regression analysis.

Linear regression analyses were performed to examine the factors affecting OT and EBL. Statistically related variables were included in the multiple linear regression analyses. The results, as shown in Table 2, indicate that preoperative HCT measurement was an independent predictor of EBL. It is intriguing that OT was longer if the patient had a narrow SA (p:0.006) and high BMI (*p*: < 0.001). However, other pelvic dimensions were found to have no significant relationship with OT.

**Table 2:** Factors affecting operation time and estimated blood loss. Operation time (*F* = 30.251, *p* (model) < 0.001, Adjusted *R*^*2*^ = 0.429); Estimated blood loss (*F* = 4.563, *p* (model) = 0.013, Adjusted *R*^*2*^ = 0.084)

**Operation time**			
**Independent variable**	**Beta**	**t**	***p***
Constant	-	1.187	0.203
BMI	0.630	7.029	< 0.001
SA	-0.244	-2.841	0.006
**Estimated blood loss**			
**Independent variable**	**Beta**	**t**	***p***
Constant	-	1.985	0.051
LC	-0.203	-1.870	0.065
Pre-operative HCT	0.239	2.198	0.031

*BMI* Body Mass Index, *HCT* Hematocrit, *LC* Lower conjugate, *SA* Symphysis angle

### Relationship between pelvic dimensions and postoperative outcomes

There was no statistically significant relationship between mortality and pelvic dimensions, and a statistically significant relationship was found only between mortality and CCI score (p:0.011). A statistically significant relationship was found between LC measurement and incidence of major complications (p:0.024), with the incidence of major complications increasing with high mean LC values. BMI and high CCI were tended to be significant for major complications. A statistically significant relationship were found to exist between PSM and ASA score, prostate volume, preoperative albumin, CCI score and ISD index (all with *p*: < 0.05) The mean ISD index measurement of patients with PSM was found to be lower than that of patients with negative surgical margins. No difference in pelvic dimensions and other variables was found among patients who were readmitted to the hospital within 30 days of surgery.

Regression analysis was used to predict minor or major complications and PSM, as shown in Table 3. No statistically significant relationship was found between pelvic dimensions and minor or major complications. Further statistical tests revealed that pathological stage, high CCI score, and ISD index measurement were independent predictors of PSM. The most striking finding was that the risk of PSM was higher in patients with low ISD index value, narrow pelvis and deep prostate apex.

**Table 3:** Multivariate analysis of various factors potentially affecting presence of complication, major complication, 90-day mortality and positive surgical margin

**Complication**	**OR (%95 CI)**	***p***
LC	0.542 (0.284–1.034)	0.063
ISD index	0.577 (0.037–8.996)	0.695
BFW index	4.868 (0.335–70.752)	0.246
BMI	1.176 (0.986–1.403)	0.072
Charlson comorbidity group (score ≥ 5)	4.197 (1.118–15.756)	0.034
ASA score	1.351 (0.443–4.122)	0.597
Clinical LN status	2.927 (0.764–11.217)	0.117
Pathological stage	1.057 (0.656–1.702)	0.821
**Major complication**	**OR (%95 CI)**	***p***
AD	2.664 (0.730–9.717)	0.138
ISD index	0.444 (0.033–5.971)	0.540
Charlson comorbidity group (score ≥ 5)	3.357 (1.009–11.169)	0.048
Clinical LN status	0.638 (0.166–2.447)	0.512
Pathological stage	1.634 (0.943–2.833)	0.080
LC	1.754 (0.889–3.463)	0.105
BMI	1.288 (1.032–1.608)	0.025
ASA score	2.664 (0.730–9.717)	0.138
**Mortality within 90 day**	**OR (%95 CI)**	***p***
AD	0.673 (0.014–32.028)	0.841
ISD index	0.066 (0.001–4.264)	0.201
LC	1.781 (0.576–5.501)	0.316
UC	1.245 (0.411–3.771)	0.698
Charlson comorbidity group (score ≥ 5)	11.492 (1.679–78.645)	0.013
BMI	1.252 (0.874–1.794)	0.220
Perioperative complication	4.492 (0.408–49.498)	0.220
Pathological stage	1.555 (0.721–3.351)	0.260
Clinical LN status	1.220 (0.146–10.229)	0.854
**Positive surgical margin**	**OR (%95 CI)**	***p***
ISD index	0.022 (0.001–0.524)	0.018
Pathological stage	12.824 (2.619–62.806)	0.002
Charlson comorbidity group (score ≥ 5)	7.563 (1.008–56.722)	0.049

*AD* Apical depth, *ASA* American Society of Anesthesiologists, *BMI* Body Mass Index, *LC* Lower conjugate, *LN* Lymph node, *SA* Symphysis angle, *UC* Upper conjugate

## Discussion

RC, is a morbid procedure with a high postoperative complication rate, frequent hospital readmissions and significant perioperative mortality risk [13]. The rate of complications within 90 days after RC and urinary diversion has been reported by various publications as 19.0–69.6% [14–17], and mortality rates have been reported in the range of 2.3–9.0% [18–21]. The overall 90-day postoperative complication and mortality rates in this study were 65.8% and 13.9%, respectively.

Only 27.8% (*n* = 22) of the patients in this study had pT2 stage BC. Furthermore, the cancer of 30.4% (*n* = 24) of the patients was upstaging to pT3 and that of 21.5% (*n* = 17) was upstaging to pT4. Thus,the it mortality rate of the patients in this study was relatively high. In a study by May et al. [22], 35% of patients diagnosed with pT1 cancer were found to have upstaging in their pathology after RC. As a result, the authors suggested that the cancer in patients in the high-risk group may be necessitating prompt operation.

A narrow, deep pelvis or the presence of the prostate at the base of the pelvis are also important considerations in the performance of a RC, such as RP, as both are linked to a higher incidence of postoperative complications and a technically challenging operation [12]. Measurements such as PD, AD, SA may play a role in manipulations during RC, such as deep dorsal vein ligation, apical dissection and the cutting of the lateral pedicles. In addition, pelvic dimensions may impact pelvic lymph node dissection in the obturator fossa [12]. Likewise, a wide and shallow pelvis may be preferable compared to a narrow and deep pelvis by surgeons [12]. All these considerations, it can be called the operative diffuculty.

Various publications have investigated whether pelvic anatomy plays a role in operative difficulty and complications of RP [7, 8, 23]. However, publications concerning the sames for RC are limited. In reference to general surgery, Boyle et al. stated that pelvic size might affect the difficulty of operation in colorectal surgery, finding that patients with smaller pelvic sizes are more likely to have PSM [24]. Likewise, Hong et al. suggested that pelvic dimensions may affect the difficulty of performing open and laparoscopic RP. The use of magnetic resonance imaging (MRI) allowed for a new measurement parameter called the pelvic dimension index (PDI) which can be used to calculate the ISD/AD ratio or ISD index in that low PDI values indicate a narrow and deep pelvis and high PDI values indicate a wide and shallow pelvis [7]. The same study found BMI to be independently associated the OT of open retropubic RP, and while pelvic size does not significantly affect the OT retropubic RP as much as patient-related factors such as BMI or prostate volume, it may have partial effect [7]. Hong et al. noted that the available working area in robot-assisted RP could be estimated using the pelvic cavity index (PCI) and that the dimensions of the pelvis did not affect the difficulty of robot-assisted RP in Korean patients [8]. Another prior study found pelvic dimensions to be correlated with OT and EBL [12]. Using, multivariate analysis, the same study determined that pelvic dimensions do not affect the difficulty of an RC but BMI and pathological stage may affect [12].

Contrary to the findings mentioned above, Mason et al. found that a deep and narrow pelvis might be associated with long OT and increased EBL in robot-assisted RP [23]. Similarly, Yao et al. reported that prostate volume/PCI ratio was a statistically significant predictor of console time and EBL in multiple linear regression analyses concluding that the OT of a procedure on a patients with large prostate volume and a small pelvis is prolonged, adding to the difficulty of the surgery [11]. The current study called these indicators as technical difficulty for RC: OT, perioperative EBL and HS. The multivariate regression analysis performed for OT, obtained results similar to those of the studies discussed above BMI and SA measurement were found to have an impact and that preoperative HCT was found to have an effect on EBL. According to the findings obtained in the present study, a narrow pelvis might cause difficulty in RC, albeit in a limited capacity.

In a study of 88 patients in a general surgery clinic, Boyle et al. found female patients with positive circumferential resection margins after colorectal surgery to have a significantly shorter ISD compared to those with negative circumferential resection margins and that preoperative measurement of ISD taken using MRI could affect the choice of adjuvant treatment [24]. A study by Matikainen et al. also involved the use of preoperative MRI to determine the effect of AD on RP. The authors reported AD to be a significant predictor of apical PSM, independent of the surgical approach and other clinicopathological variables [9]. In the current study, we found pathological T stage, high CCI score and ISD index to be independent risk factors for PSM. This result may be explained by the fact that the risk of PSM is higher in patients with a small ISD index value, narrow pelvis and deep prostate apex.

Surgical site infection (SSI) is a widespread postoperative complication that causes significant pain and suffering. SSI associated with negative economic effects, increased morbidity, extended postoperative HS, readmission, sepsis, and death [25]. Previous research has established that postoperative SSI is diagnosed in (21.8%) cases [25]. Moreover, SSIs following a procedure to treat colorectal cancer has been found to be significantly more common among patients that are older (> 70 years), obese (≥ 30 kg/m2), have ASA scores ≥ 3, have diabetes and with a history of chronic steroid use, undergoing open, dirty or contaminated surgery [25]. In our study, SSI was observed in 12 patients (15.1%).

Thus, evaluating a patient’s pelvic anatomy before a RC may help predict postoperative results, allowing for preparation for the possible difficulties of the procedure and more successful outcomes.

### Limitations of the study

The limitations of the present study include its retrospective nature, short follow-up times and relatively small sample size.

## Conclusion

The aim of this research was to examine preoperative predictions of postoperative complications in patients undergoing RC. The primary finding of this study was that SA is an independent predictor of OT, suggesting that surgeons may experience more difficulty performing a RC procedure on the patients with a narrow SA.

The second major finding was that the risk of PSM increased in patients with a narrow and deep pelvis. This suggests that ISD/AD ratio is a significant predictor of PSM and this relationship is independent of patient-related and other clinicopathological variables. Furthermore, the results of this study suggest that other anatomical variations in the pelvis may not be essential factors in terms of operative difficulty or minor or major complications. While patient-related features, such as CCI score and BMI, may increase mortality. This is the first study to present evidence for the pelvic dimensions of PSM in RC.

## Supplementary Information

**Additional file 1: Supplementary Table 1**. Incidence of postoperative complications.

## Data Availability

The datasets used and/or analysed during the current study available from the corresponding author on reasonable request.

## Abbreviations

AD: Apical depth
ASA: American Society of Anesthesiologists
BFW: Bony femoral width
BMI: Body mass index
BC: Bladder cancer
CCI: Charlson comorbidity index
CT: Computed tomography
EBL: Estimated blood loss
HCT: Hematocrit
HS: Hospital stay
ISD: Interspinous distance
LC: Lower conjugate
MRI: Magnetic resonance imaging
OT: Operative time
PD: Pelvic depth
PDI: Pelvic dimension index
PSM: Positive surgical margin
RC: Radical cystectomy
RP: Radical prostatectomy
SW: Soft tissue width
SA: Symphysis angle
SSI: Surgical site infection
UC: Upper conjugate
