# Supplementary material for: Can pelvic diameter measurement have an effect on surgical outcomes in radical cystectomy?
Source: BMC Urol. 2023 Jun 7;23:105. doi: 10.1186/s12894-023-01277-8 (PMC10246386; doi:10.1186/s12894-023-01277-8)
Supplement: Supplementary file 1 — Additional file 1: Supplementary Table 1. Incidence of postoperative complications. [file 12894_2023_1277_MOESM1_ESM.docx]

**Supplementary Table1**. Incidence of postoperative complications (within 90 days).

|  | **n** |
| --- | --- |
| **Gastrointestinal** | 28 (35,4%) |
| İleus | 21 |
| Emesis | 4 |
| İleal anastomotic leak | 3 |
| **Infection** | 22 (27,8%) |
| Urinary tract infection | 12 |
| Pyelonephritis | 5 |
| Urosepsis | 5 |
| **Genitourinary** | 9 (11,4%) |
| Renal failure | 9 |
| **Cardiac** | 7 (8,9%) |
| Arrhythmia | 5 |
| Hypotension | 2 |
| **Pulmonary** | 8 (10,1%) |
| Pneumonia | 3 |
| Pleural effusion | 3 |
| Respiratory distress | 1 |
| Pulmonary embolism | 1 |
| **Hematological/Vascular** | 11 (13,9%) |
| Anemia requiring transfusion | 8 |
| Deep venous thrombosis | 2 |
| Disseminated intravascular coagulation | 1 |
| **Wound/Skin** | 17 (21,5%) |
| Wound infection | 12 |
| Wound dehiscence | 5 |
| Fascial evisceration | 1 |
| **Neurological** | 3 (3,8%) |
| Confusion | 3 |
| **Other** | 1 (1,3%) |
| Lymphocele | 1 |
